# Crizotinib Inhibits Viability, Migration, and Invasion by Suppressing the *c-Met*/*PI3K*/*Akt* Pathway in the Three-Dimensional Bladder Cancer Spheroid Model

**DOI:** 10.3390/curroncol32040236

**Published:** 2025-04-17

**Authors:** Byeongdo Song, Danhyo Kim, Jin-Nyoung Ho, Van-Hung Le, Sangchul Lee

**Affiliations:** 1 Department of Urology, Hanyang University Guri Hospital, 153, Gyeongchun-ro, Guri-si 11923, Gyeonggi-do, Republic of Korea; 2236930@hyumc.com; 2 Department of Urology, Seoul National University Bundang Hospital, 166, Gumi-ro, Bundang-gu, Seongnam-si 13620, Gyunggi-do, Republic of Korea; r2430@snubh.org (D.K.); 97875@snubh.org (J.-N.H.); hunglv24@vinmec.com (V.-H.L.); 3 Department of Urology, Vinmec International Hospital, 458 P. Minh Khai, Hanoi 100000, Vietnam; 4 Department of Urology, Seoul National University College of Medicine, 71, 101 Daehak-ro, Jongno-gu, Seoul 03080, Republic of Korea

**Keywords:** crizotinib, 3D cell culture, spheroid, cisplatin-resistant bladder cancer, bladder cancer

## Abstract

We aimed to evaluate the therapeutic potential of crizotinib, a broad-spectrum tyrosine kinase inhibitor against bladder cancer (BC) cells, based on a three-dimensional (3D) cell culture system. After proliferating cell masses (spheroids) using T24 cisplatin-naïve and T24R2 cisplatin-resistant human BC cell lines, the spheroids were exposed to various crizotinib concentrations in order to derive an ideal crizotinib concentration to suppress cell survival, migration, and invasion. Crizotinib suppressed cell proliferation, migration, and invasion in both T24 and T24R2 BC cell lines under a 3D spheroid model, which was more appropriate than the conventional two-dimensional cell culture model. Real-time quantitative polymerase chain reaction analysis revealed a reduced expression of *E-cadherin* and an enhanced expression of *vimentin*, suggesting EMT suppression and the subsequent suppression of tumor aggressiveness following crizotinib administration. Meanwhile, the expressions of apoptosis-related genes increased. Western blot analysis revealed that the expression levels of *phosphorylated mesenchymal–epithelial transition factor (c-Met)* and *phosphorylated Akt* decreased following crizotinib administration, suggesting that the antitumor effect of crizotinib can be associated with the inhibition of the phosphorylated activation of the *c-Met*/*PI3K*/*Akt* pathway. Crizotinib showed a potential antitumor effect on both cisplatin-naïve and cisplatin-resistant human BC cells, likely through *c-Met*-induced *PI3K*/*Akt* pathway inhibition.

## 1. Introduction

Bladder cancer (BC) is one of the most frequent malignancies worldwide, ranking 4th and 11th as the leading cancer among males and females, respectively [1]. During initial diagnosis, approximately 70–75% of patients have non-muscle-invasive BC (NMIBC), whereas the remaining patients have muscle-invasive BC (MIBC) [2]. Although NMIBC is frequently treated by transurethral resection followed by intravesical therapy [3], radical cystectomy plus pelvic node dissection has been the gold standard treatment option for MIBC. However, these treatments are frequently insufficient for MIBC, partially owing to the risk of micrometastasis [4], with a 5-year survival rate of 20–40% and a 3-year metastasis rate of approximately 50% [5].

Therefore, cisplatin-based neoadjuvant chemotherapy, including dose-dense methotrexate, vinblastine, doxorubicin, and cisplatin or gemcitabine and cisplatin regimens followed by radical cystectomy, has been the standard of care for localized MIBC [6]. Meanwhile, for advanced BC, immune checkpoint inhibitors (ICIs) have become an additional second-line treatment option [7].

Although cisplatin-based combination therapy has achieved relatively high objective response rates of 36–65% [8], drug resistance [9] and considerable adverse effects (e.g., renal dysfunction) are significant obstacles to successful treatment [10], with a median survival of <1 year [11,12].

Over the last decade, ICIs have revolutionized the therapeutic landscape of BC. The first ICI was ipilimumab, a cytotoxic T-lymphocyte-associated antigen 4 inhibitor that was approved in the United States in 2011, whereas nivolumab and pembrolizumab [13], which are programmed cell death protein 1 inhibitors, were approved for urothelial carcinoma treatment in 2014 [14,15,16]. Recently, novel agents, including erdafitinib, a *fibroblast growth factor receptor (FGFR) tyrosine kinase inhibitor*, and enfortumab vedotin, *an antibody–drug conjugate targeting nectin-4* [17], have been introduced in the clinical field. For cisplatin-ineligible patients with BC, atezolizumab [18] and pembrolizumab [13] have shown acceptable antitumor outcomes and tolerability. However, the response rate to ICI monotherapy in BC is modest [19,20,21,22,23]. Therefore, a novel target is required for the treatment of BC, especially cisplatin-resistant BC.

The expression of *mesenchymal–epithelial transition factor (c-Met)*, one of the tyrosine kinase receptors, has been reported to be strongly associated with BC development and prognosis, suggesting that *c-Met* could be a potential target for BC treatment [24]. Previous studies have reported that *c-Met* signaling is enhanced in various chemo-resistant cell lines, suggesting that targeting *c-Met* can reverse chemotherapy resistance [25]. Moreover, *c-Met*/*Pyk2* pathway inhibition has been suggested as a method for overcoming *FGFR* inhibition resistance [26].

Crizotinib is a small-molecule oral selective inhibitor of *anaplastic lymphoma kinase (ALK)*, *c-Met*/*hepatocyte growth factor (HGF) receptor*, and *ROS1 receptor kinase*, which has been approved by the Food and Drug Administration (FDA) for the treatment of *ALK* rearrangement that is positive with metastatic non-small-cell lung cancer (NSCLC) [27]. In addition to NSCLC, the antitumor effect of crizotinib has been reported in advanced anaplastic lymphoma [28], gastric cancer [29], cervical cancer [30], clear cell renal cell carcinoma [31], and prostate cancer [32]. However, studies evaluating the therapeutic effect of crizotinib for BC are limited. To the best of our knowledge, only one study has demonstrated the potential antitumor effect of crizotinib at the cellular level [33]; however, this study did not evaluate the therapeutic effect in cisplatin-resistant human BC cell lines.

Conventionally, in vitro cellular evaluations have been performed using a two-dimensional (2D) culture system. However, over the last decade, the three-dimensional (3D) spheroid culture system has become a useful platform in drug screening, showing a better ability to reproduce natural environments at the cellular level than the conventional 2D culture system [34].

Here, we aimed to investigate the antitumor effect of crizotinib against cisplatin-naïve and cisplatin-resistant human BC cell lines at the cellular level using the 3D spheroid culture system.

## 2. Materials and Methods

### 2.1. Cell Lines and Reagents

The T24 cell line was purchased from the American Type Culture Collection (Manassas, VA, USA). The cisplatin-resistant cell line T24R2 was established through the serial desensitization of T24 cells, showing resistance to cisplatin (2 μg/mL) [35]. Cells were cultured in an RPMI-1640 medium (Gibco; Invitrogen, Carlsbad, CA, USA) supplemented with 10% heat-inactivated fetal bovine serum (FBS) and 1% penicillin/streptomycin (Gibco; Invitrogen) in a humidified atmosphere of 95% air and 5% CO_2_ at 37 °C. Crizotinib was obtained from Selleck Chemicals (cat. no. S1068; Houston, TX, USA).

### 2.2. Spheroid Formation

For spheroid formation, 2.5 × 10^3^ BC cells were seeded in 96-well spheroid microplates (cat. no. CLS4515; Corning, New York, NY, USA) and were subsequently cultured in an RPMI-1640 medium for 4 days, as previously reported [36]. BC spheroids of approximately 230–280 μm were formed and treated with crizotinib for 24 h.

### 2.3. Cell Counting Kit (CCK)-8 Assay

The cell viability of each monolayer and spheroid was evaluated utilizing the CCK-8 assay. The monolayers and spheroids were administered with crizotinib at a variety of concentrations (0, 5, 10, 20, and 50 μM) for 24 h. A total of 10 μL of the CCK-8 reagent (cat. no. CK04; Dojindo Molecular Technologies, Gaithersburg, MD, USA) was added to every well, which was subsequently incubated for 4 h. Spectrophotometric absorbance was assessed utilizing a microplate reader (Molecular Devices, Sunnyvale, CA, USA) at 450 nm.

### 2.4. Live/Dead Staining

BC spheroid viability was measured using the LIVE/DEAD Viability/Cytotoxicity Kit (cat. no. L3224; Invitrogen; Thermo Fisher Scientific, Inc., Waltham, MA, USA) following the manufacturer’s instructions. The spheroids were stained with a mixture of live cell staining dye, dead cell staining dye, and nuclei staining dye Hoechst 33342 (cat. no. 62249; Thermo Fisher Scientific, Inc.) and were subsequently incubated for 30 min. Cell staining was visualized using a fluorescent microscope.

### 2.5. Matrigel Migration Assay

For the migration assay, 25 μL of the Matrigel matrix (cat. no. 356234; 1 mg/mL, BD Bioscience, San Jose, CA, USA) was precoated into the upper chamber (cat. no. 3422; 6.5 mm insert, 8.0 µm pore size; Corning Costar, Corning, NY, USA) before adding the FBS-free RPMI-1640 containing the spheroids. Meanwhile, the lower chamber was loaded with 700 μL of culture media containing 10% FBS and crizotinib at a variety of concentrations (0, 5, 10, 20, and 50 μM). After incubation for 24 h, the migrated cells were stained utilizing the LIVE/DEAD Viability/Cytotoxicity Kit (Invitrogen). Images of the migrated cells were obtained under a microscope (Thunder Imager; Leica LAS X core 7.7.4 software; magnification, 100×, Wetzlar, Germany). To measure the migrated area, ImageJ software (National Institutes of Health, Bethesda, MD, USA; https://imagej.nih.gov/ij/download.html, accessed on 15 April 2025) was used.

### 2.6. Collagen Invasion Assay

For the 3D invasion assay, the spheroids were embedded in Cultrex 3D culture matrix rat collagen type I gel (cat. no. 3447-020-01; R&D systems Inc., Minneapolis, MN, USA) and were subsequently incubated for 30 min. A medium which contains 10% FBS and crizotinib (0, 5, 10, and 20 μM) was administered to the collagen gel. After an incubation period of 24 h, the invaded cells were stained using the LIVE/DEAD Viability/Cytotoxicity Kit (Invitrogen). Images of the invading cells were obtained using a microscope (Thunder Imager). ImageJ software (National Institutes of Health) was used for measuring the invaded area.

### 2.7. Quantitative Real-Time Polymerase Chain Reaction (qPCR)

The total RNA (500 ng) of the spheroids was extracted utilizing the RNeasy Mini-Kit (cat. no. 74104; Qiagen, Maryland, MD, USA) and was subsequently quantified using the NanoDrop ND 1000 (Thermo Fisher Scientific, Inc.). The complementary DNA (cDNA) was synthesized utilizing the iScript cDNA Synthesis Kit (cat. no. 1708891; Bio-Rad, Hercules, CA, USA) following instructions of the manufacturer. Each reaction included 2 μL of cDNA, 2 μL of primer (F = 1, R = 1), 10 μL of Power SYBR Green PCR Master Mix (cat. no. 4367659; Applied Biosystems, Warrington, UK), and 6 μL of H_2_O (final volume: 20 μL). Real-time PCR cycles comprised 2 min at 50 °C, 10 min at 95 °C for the hold stage, 40 cycles of 15 s at 95 °C, and 1 min at 60 °C. The *GAPDH* level was used as an endogenous control. The expressions of target genes were estimated using the 2^−ΔΔCt^ method. The PCR primers are listed in Table 1.

### 2.8. Western Blot Analysis

Cells were lysed using an RIPA cell lysis buffer containing protease inhibitor. Cell lysates were loaded and separated in 8–12% SDS-PAGE and were subsequently transferred to polyvinylidene fluoride membranes (Bio-Rad, Berkeley, CA, USA). The membrane was blocked with 5% skimmed milk at room temperature for 1 h and was incubated with primary antibodies (*Akt, phosphorylated Akt [Ser473]*, *c-Met, phosphorylated c-Met [Y1234/1235]*, *phosphoinositide 3-kinase [PI3K]*, *phosphorylated PI3K [Tyr485]/p55 [Tyr199]*, and *β-actin* [Cell Signaling Technology Inc., Beverly, MA, USA]) overnight at 4 °C. Subsequently, proteins were visualized using enhanced chemiluminescence (cat. no. RPN2232; Amersham, UK). The protein intensity was analyzed using ImageJ software (National Institutes of Health). The original images of the Western blot analysis showing all bands with all molecular weight markers are illustrated in Supplementary Figure S1.

### 2.9. Statistical Analysis

All statistical analyses were performed using IBM Statistical Package for the Social Sciences version 27.0 (IBM Corp., Armonk, NY, USA). Data were expressed as means ± standard deviations of three independent experiments. Statistical significance was defined as a two-tailed *p*-value < 0.05 using analysis of variance followed by Tukey’s multiple-range test.

## 3. Results

### 3.1. Crizotinib Inhibits the Cell Viability of BC Cells and Spheroids

**Figure 1:**
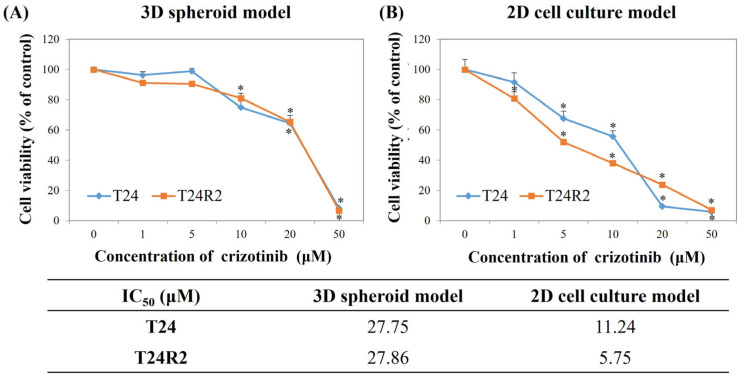
Comparison of cell viability and the 50% inhibitory concentration of cell growth (IC50) value of crizotinib in T24 and T24R2 human BC cell lines under the three-dimensional spheroid model (**A**) and two-dimensional cell culture model (**B**). Bars represent the mean ± standard deviation of triplicate samples from three independent experiments; asterisks (*) indicate statistically significant differences (*p* < 0.05). Some error bars may be too small to be visible.

T24 and T24R human BC cell lines were exposed to crizotinib at different concentrations (5, 10, 20, and 50 μM), and the cell viability was assessed using the CCK-8 assay. Treatment with crizotinib for 24 h inhibited BC cell and spheroid proliferation in a concentration-dependent manner (Figure 1). The 50% inhibitory concentration of cell growth (IC50) value of crizotinib was twofold higher for T24 (27.75 vs. 11.24 μM) and fivefold higher for T24R2 in 3D spheroids than in the 2D culture (27.86 vs. 5.75 μM) (Figure 1).

**Figure 2:**
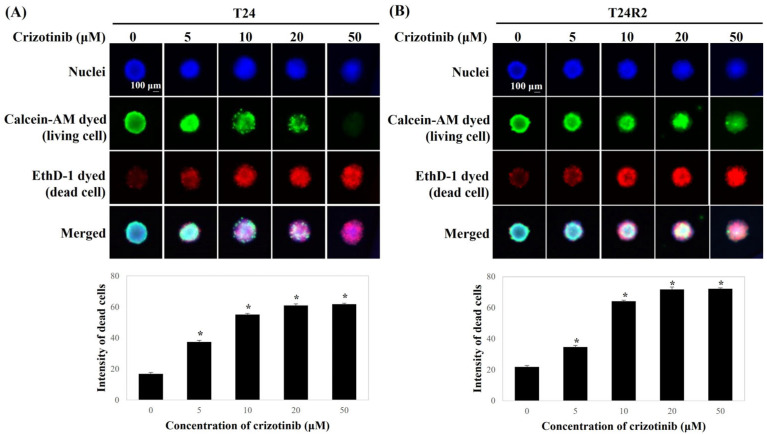
Comparison of cell viability of T24 (**A**) and T24R2 human BC cell lines (**B**) according to crizotinib concentration using the LIVE/DEAD Viability/Cytotoxicity Kit under the three-dimensional spheroid model. Bars represent the mean ± standard deviation of triplicate samples from three independent experiments; asterisks (*) indicate statistically significant differences (*p* < 0.05). Some error bars may be too small to be visible.

Moreover, the viability of the BC spheroids was measured using the LIVE/DEAD Viability/Cytotoxicity Kit. Crizotinib treatment increased the fluorescence intensity of the dead cells (red-stained cells) of the BC spheroids in a dose-dependent manner (Figure 2).

### 3.2. Crizotinib Inhibits the Migration of BC Spheroids

**Figure 3:**
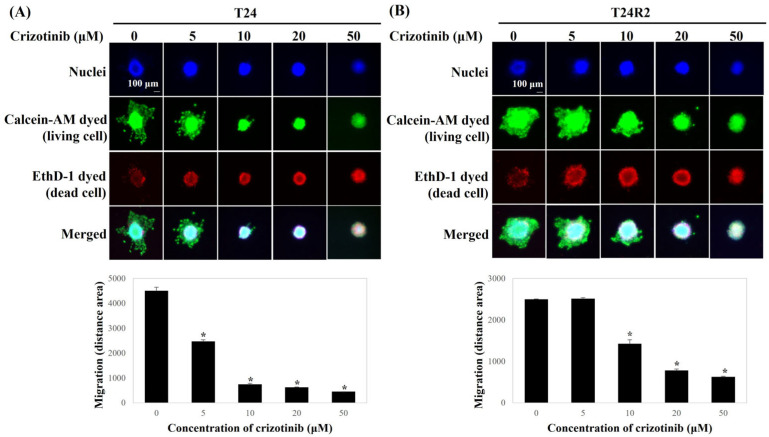
Comparison of the dose-dependent effect of crizotinib on cell migration under the three-dimensional spheroid model for T24 (**A**) and T24R2 human BC cell lines (**B**). Bars represent the mean of triplicate samples ± SD. Data are representative of three independent experiments; asterisks (*) indicate statistically significant differences from the control group (*p* < 0.05). Some error bars are too small to be visible.

Compared with the control, the migrated area of BC spheroids on the matrix-coated membrane in the presence of crizotinib was significantly reduced (Figure 3). The migration trend was rarely observed in T24 and T24R2 cells following crizotinib treatment at a concentration of >20 μM.

### 3.3. Crizotinib Inhibits the Invasion of BC Spheroids

**Figure 4:**
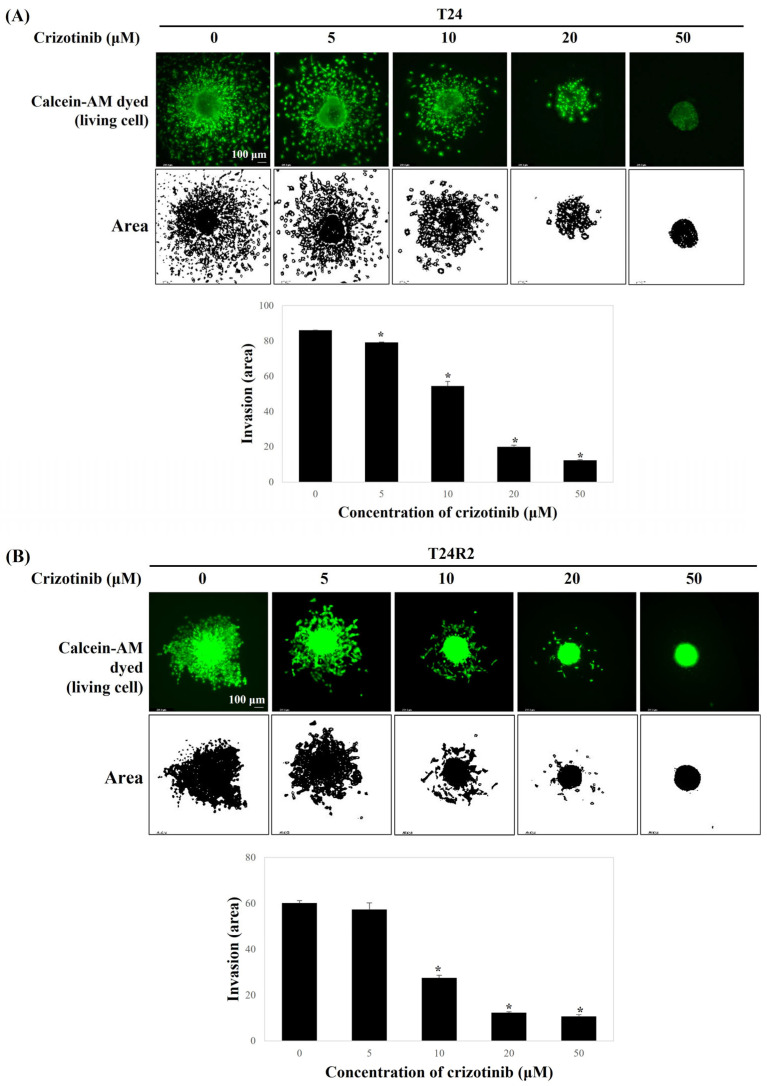
Comparison of the dose-dependent effect of crizotinib on cell invasion under the three-dimensional spheroid model for T24 (**A**) and T24R2 human BC cell lines (**B**). Bars represent the mean ± standard deviation of triplicate samples from three independent experiments; asterisks (*) indicate statistically significant differences (*p* < 0.05). Some error bars may be too small to be visible.

A trans-well assay was performed to examine the effect of crizotinib in the invasion of BC spheroids. The invaded area of BC spheroids embedded in the collagen matrix was estimated using ImageJ. As crizotinib is administered in increasing doses from 5 μM to 50 μM, crizotinib statistically significantly inhibited BC spheroid invasion in a dose-dependent manner. The invasions of both T24 and T24R2 BC spheroids into the surrounding collagen matrix were significantly inhibited at 5 μM of crizotinib (Figure 4). In particular, almost no invasion occurred in the spheroid treated with 50 μM crizotinib.

### 3.4. Crizotinib Suppresses EMT- and Induces Apoptosis-Related Gene Expression of BC Spheroids

**Figure 5:**
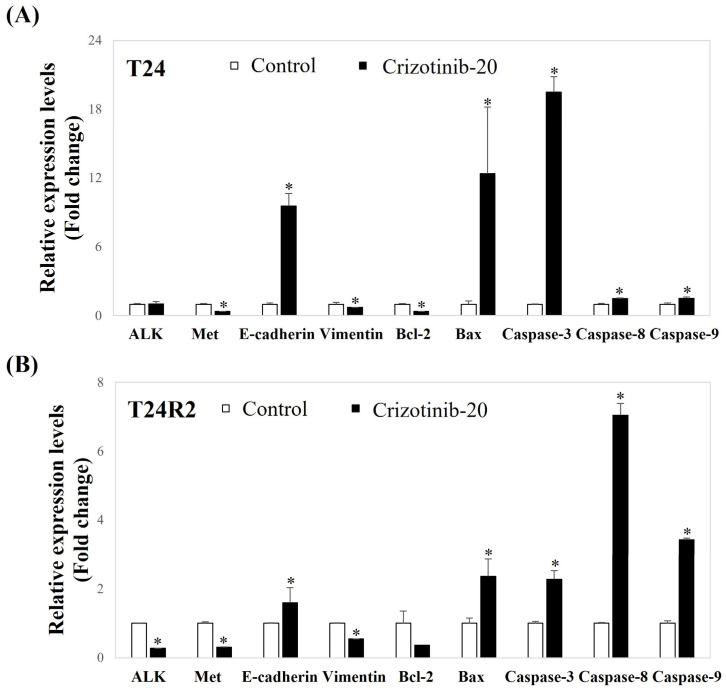
Comparison of the effect of crizotinib on the expression of EMT- and apoptosis-related genes using quantitative real-time polymerase chain reaction analysis in T24 (**A**) and T24R2 human BC cell line spheroids (**B**). Both cell line spheroids were treated with 10% FBS to serve as controls or were treated with 20 μM of crizotinib for 24 h. Bars represent the mean ± standard deviation of triplicate samples from three independent experiments; asterisks (*) indicate statistically significant differences (*p* < 0.05). Some error bars may be too small to be visible.

Figure 5 demonstrates the measured mRNA expression rate of each EMT- and apoptosis-related gene in both T24 and T24R2 BC spheroids according to qPCR. The mRNA expression of the mesenchymal marker *vimentin* was downregulated, whereas that of the epithelial marker *E-cadherin* was significantly upregulated in crizotinib-treated BC spheroids. These results demonstrate that crizotinib suppressed the EMT of BC spheroids (Figure 5).

In addition, we also investigated the mRNA expression of apoptosis-related genes using qPCR in BC spheroids. Crizotinib treatment markedly decreased the *Bcl-2*/*Bax* ratio. Moreover, the expressions of *cleaved caspase-3*, *-8*, and *-9* mRNA, which are apoptosis-related genes, were significantly increased in crizotinib-treated BC spheroids in comparison with the control (Figure 5).

### 3.5. Crizotinib Inhibits the c-Met/PI3K/Akt Pathway in BC Spheroids

**Figure 6:**
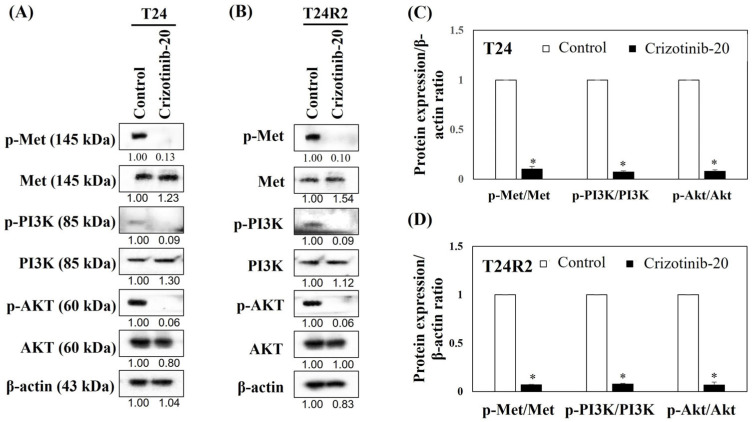
Crizotinib inhibits the *c-Met*/*PI3K*/*Akt* signaling pathway in T24 (**A**) and T24R2 human BC cell line spheroids (**B**). Both cell line spheroids were treated with 10% FBS to serve as controls or were treated with 20 μM of crizotinib for 24 h. The suppression of each protein that is related to the *c-Met*/*PI3K*/*Akt* signaling pathway after the administration of crizotinib was quantified, as shown in the histogram, in T24 (**C**) and T24R2 human BC cell lines (**D**). Bars represent the mean ± standard deviation of triplicate samples from three independent experiments; asterisks (*) indicate statistically significant differences (*p* < 0.05). Some error bars may be too small to be visible.

To identify the anti-cancer mechanism of crizotinib, we measured the expressions of *c-Met*, *PI3K*, and *Akt* in T24 and T24R2 BC spheroids after the administration of crizotinib using Western blot analysis. When treating the spheroids of cisplatin-sensitive T24 and cisplatin-resistant T24R2 cells with 20 µM crizotinib, the expression rates of *phosphorylated c-Met*, *PI3K*, and *Akt* significantly decreased (Figure 6).

## 4. Discussion

Our study demonstrated that crizotinib could suppress the proliferation, migration, and invasion of cisplatin-naïve and cisplatin-resistant BC cell lines. As the incidence of *ALK* mutations in urothelial carcinoma is scarce, the potentiality of *ALK* inhibitors as therapeutic agents for urothelial carcinoma is considered to be limited [37]. Although crizotinib has been known as a potent ALK inhibitor that has been approved by the FDA for patients with advanced *ALK*-positive NSCLC [27], it also inhibits various tyrosine kinases, including *ROS1*, *c-Met*, and *RON* kinases [38]. Several previous studies have reported the potential of crizotinib as a therapeutic agent for several malignancies; however, only one previous study has evaluated the antitumor effect of crizotinib against BC [33].

In our study, under a 2D culture environment, crizotinib suppressed cell viability, with an IC50 value of 11.24 and 5.75 μM for the T24 and T24R2 cell lines, respectively. Meanwhile, under a 3D spheroid culture, both T24 and T24R2 cell lines became less sensitive to crizotinib, with IC50 values of 27.75 and 27.86 μM, respectively. Such results were correlated with those of previous studies, which reported that IC50 values increased in the 3D spheroid culture compared with that in the 2D culture [39,40,41]. For gastric cancer cells, the 3D spheroid cell model showed higher IC50 values [42] that were approximately twice as high as those in the conventional 2D cell culture model [43].

Considering that Berrouet et al. [44] suggested that the 3D spheroid culture model is more appropriate for evaluating IC50 than the conventional 2D culture model, our IC50 results using the 3D spheroid culture model could be more appropriate for determining ideal drug dosages for future in vivo animal experiments and further clinical trials.

The T24 and T24R2 cell lines showed higher expression levels of *E-cadherin*, *Bax*, and *caspase-3*, *-8*, and *-9*, as well as lower expression levels of *ALK*, *c-Met*, vimentin, and *Bcl-2* compared to the crizotinib-naïve status. The reduced expression of E-cadherin, an epithelial marker of EMT, and the enhanced expression of vimentin, a mesenchymal marker of EMT, are features of high-stage and high-grade tumors in BC [45]. *Bcl-2* and *Bax* are two significant regulator genes in the mitochondrial apoptotic pathway [46]. The *Bcl-2* gene is suggested to suppress apoptotic cell death, whereas *Bax*, an essential homolog of *Bcl-2*, is a promoter of apoptosis [47]. Moreover, *caspase-3*, *-8*, and *-9* play a major role in cell apoptosis [48,49]. Therefore, our qPCR results are correlated with those of previous studies [45,46,47,48,49], suggesting that crizotinib can present the antitumor effect by inducing cell apoptosis and suppressing EMT.

Meanwhile, Western blot analysis results revealed that crizotinib inhibits the expression and activation of *c-Met*, *PI3K*, and *Akt* in T24 and T24R2 cell lines. Considering that *ALK* genomic alterations are rare and probably without prognostic implications in urothelial carcinoma [37], crizotinib may show antitumor activity against cisplatin-naïve and cisplatin-resistant BC by inhibiting the *c-Met*/*PI3K*/*Akt* pathway rather than the *ALK* pathway. The *PI3K*/*Akt* signaling axis is a major arm of the *c-Met* signaling pathway, which is involved in the cell survival pathway [50]. Therefore, targeting the *PI3K*/*Akt* signaling pathway has been suggested as a potential therapeutic strategy for advanced BC [51]. Furthermore, Lee et al. [33] suggested that the antitumor effect of crizotinib can be induced by inhibiting the *HGF*-activated phosphorylation of *c-Met*, *Akt*, and *Erk*.

Moreover, our results showed no difference in *c-Met*/*PI3K*/*Akt* pathway activation and IC50 values of crizotinib between the T24 and T24R2 cell lines under the 3D spheroid model, suggesting that crizotinib can suppress BC cell lines regardless of cisplatin sensitivity. A previous study suggested that activating the *PI3K*/*Akt*/*Bcl-2* signaling pathway can contribute to the cisplatin resistance of BC [52]. However, considering that Lee et al. [33] reported that no BC cell lines produced detectable *HGF* levels, suggesting that autocrine *HGF* is rare in BC, the paracrine activation of the *c-Met*/*PI3K*/*Akt* pathway may be involved in the in vivo environment. To evaluate whether the paracrine activation of the *c-Met*/*PI3K*/*Akt* pathway in cisplatin-resistant BC can affect the function of crizotinib, further in vivo studies should be conducted.

This study had some limitations. In this study, only the T24 and T24R2 cell lines were used for evaluating the antitumor effect of crizotinib, thereby requiring further in vitro and in vivo studies. Considering that *c-Met* amplification showed a high sensitivity for crizotinib in NSCLC cell lines but no sensitivity for cisplatin [53], other BC cell lines should be included for evaluating the association between *c-Met* amplification level and crizotinib sensitivity. Lastly, to identify the presence of the paracrine pathway in *c-Met*/*PI3K*/*Akt* pathway activation and the therapeutic effect of crizotinib in cisplatin-resistant BC, further in vivo studies are warranted.

Despite these limitations, to the best of our knowledge, this is the first study to evaluate the antitumor effect of crizotinib against cisplatin-naïve and cisplatin-resistant BC cell lines using the 3D spheroid culture model.

## 5. Conclusions

This study demonstrates the therapeutic potential of crizotinib in cisplatin-naïve and cisplatin-resistant BC under the 3D spheroid model, which presented more appropriate IC50 values than the conventional 2D cell culture model. Therefore, crizotinib is considered a potential therapeutic agent for the treatment of BC.

## Figures and Tables

**Table 1:** Quantitative real-time polymerase chain reaction primers used in this study.

Gene	Primer 5′→3′
*ALK* (F)	AAA GAA ACC CAC AGC TGC AG
*ALK* (R)	TAA ACC AGG AGC CGT ACG TT
*c-Met* (F)	ATA CGG TCC TAT GGC TGG TG
*c-Met* (R)	TTG AAA TGG TTT GGG CTG GG
*E-cadherin* (F)	CAG CAC GTA CAC AGC CCT AA
*E-cadherin* (R)	ACC TGA GGC TTT GGA TTC CT
*Vimentin* (F)	GTT TCC AAG CCT GAC CTC AC
*Vimentin* (R)	GCT TCA ACG GCA AAG TTC TC
*Bax* (F)	AGA CAG GGG CCT TTT TGC TA
*Bax* (R)	AAT TCG CCG GAG ACA CTC G
*Bcl-2* (F)	CTT TGA GTT CGG TGG GGT CA
*Bcl-2* (R)	AGT TCC ACA AAG GCA TCC CA
*Caspase-3* (F)	AAG ATA CCG GTG GAG GCT GA
*Caspase-3* (R)	AAG GGA CTG GAT GAA CCA CG
*Caspase-8* (F)	TCT GGA GCA TCT GCT GTC TG
*Caspase-8* (R)	CCT GCC TGG TGT CTG AAG TT
*Caspase-9* (F)	GGC TGT CTA CGG CAC AGA TGG A
*Caspase-9* (R)	CTG GCT CGG GGT TAC TGC CAG
*GAPDH* (F)	TGC ACC ACC AAC TGC TTA G
*GAPDH* (R)	AGA GGC AGG GAT GAT GTT C

## Data Availability

The data generated in the present study may be requested from the corresponding author.

## Supplementary Materials

The following supporting information can be downloaded at: https://www.mdpi.com/article/10.3390/curroncol32040236/s1, Figure S1: The image of whole blot (uncropped blots) showing all bands with all molecular weight markers on the Western blot assay.

## Abbreviations

The following abbreviations are used in this manuscript:

3D	Three-dimensional
2D	Two-dimensional
c-Met	Mesenchymal–epithelial transition factor
BC	Bladder cancer
NMIBC	Non-muscle-invasive bladder cancer
MIBC	Muscle-invasive bladder cancer
ICI	Immune checkpoint inhibitor
FGFR	Fibroblast growth factor receptor
ALK	Anaplastic lymphoma kinase
HGF	Hepatocyte growth factor
FDA	Food and Drug Administration
CCK	Cell counting kit
qPCR	Quantitative real-time polymerase chain reaction
NSCLC	Non-small-cell lung cancer
FBS	Fetal bovine serum
