# Supplementary figures and images for: Crizotinib Inhibits Viability, Migration, and Invasion by Suppressing the c-Met/PI3K/Akt Pathway in the Three-Dimensional Bladder Cancer Spheroid Model
Source: Curr Oncol. 2025 Apr 17;32(4):236. doi: 10.3390/curroncol32040236 (PMC12025888; doi:10.3390/curroncol32040236)

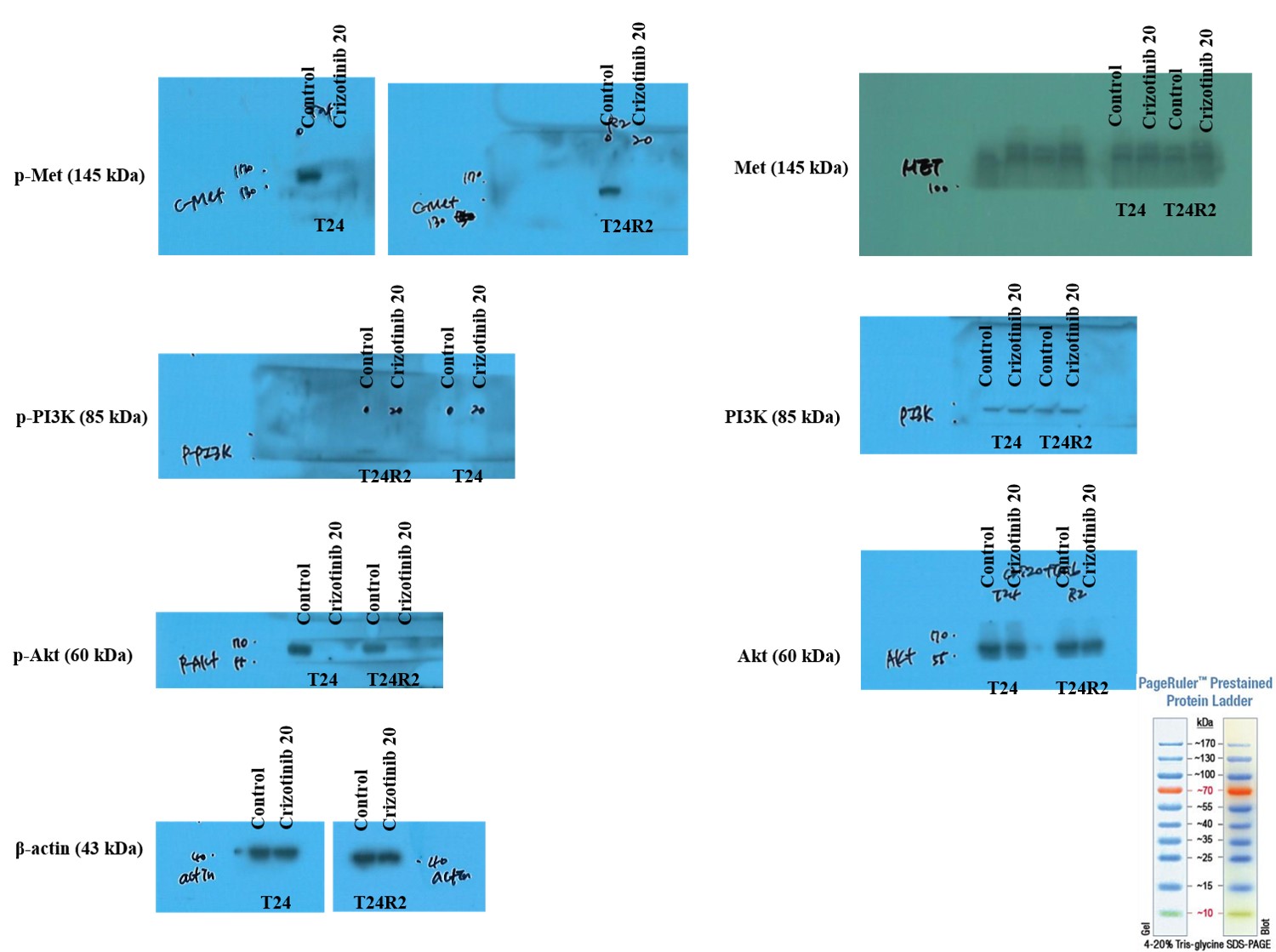

Supplement: Supplementary file 1 [file curroncol-32-00236-s001.zip › curroncol-3470101-supplementary.jpg]
